# Evaluation of neuropathological effects of a high‐fat high‐sucrose diet in middle‐aged male C57BL6/J mice

**DOI:** 10.14814/phy2.13729

**Published:** 2018-06-11

**Authors:** Bradley J. Baranowski, Kirsten N. Bott, Rebecca E. K. MacPherson

**Affiliations:** ^1^ Department of Health Sciences Brock University Ontario Canada

**Keywords:** Aging, Alzheimer's disease, inflammation, insulin resistance, obesity

## Abstract

Metabolic dysfunction related to diet‐induced obesity has recently been linked to the pathogenesis of sporadic Alzheimer's disease (AD). However, the underlying mechanisms linking obesity and AD remain unclear. The purpose of this study was to examine early alterations in brain insulin signaling, inflammatory/stress markers, and energetic stress in a model of diet‐induced obesity during middle age. Male C57BL/6J mice were randomized to either a control diet (AGE *n* = 12) or high‐fat and sucrose diet (AGE‐HFS *n* = 12) for 13‐weeks from 20‐weeks of age. Prefrontal cortex and hippocampal samples were collected at 20‐weeks of age (BSL *n* = 11) and at 33‐weeks of age (AGE and AGE‐HFS). The HFS diet resulted in increased body weight (30%; *P* = 0.0001), increased %fat mass (28%; *P *= 0.0001), and decreased %lean mass (33%; *P *= 0.0001) compared to aged controls. In the prefrontal cortex, AGE‐HFS resulted in increased 5′ adenosine monophosphate – activated protein kinase (AMPK) phosphorylation (*P *= 0.045). In the hippocampus, AGE‐HFS resulted in increased extracellular signal‐regulated kinase (ERK) and c‐Jun N‐terminal kinase (JNK) phosphorylation and protein kinase B (Akt) serine473 and glycogen synthase kinase (GSK) phosphorylation (*P *< 0.05). Results from this study demonstrate that aging combined with a HFS diet results in increased inflammation (pERK and pJNK) and energetic stress (pAMPK) in the hippocampus and prefrontal cortex, respectively. Together these novel results provide important information for future targets in early AD pathogenesis.

## Introduction

Alzheimer's disease (AD) is a progressive neurodegenerative disorder resulting in cognitive decline later in life. Aging is the largest risk factor for AD (Jorm et al. 1987), however diet‐induced obesity is directly implicated in the etiology of AD (Lester‐Coll et al. 2006; Verdelho et al. 2007; Maesako et al. 2012a,b, 2013; Bhat and Thirumangalakudi 2013). A recent analysis of obesity trends demonstrated that middle‐aged Americans have the highest obesity rate of any age group at 41.0% (Flegal et al. 2016), and research has proven that obesity in middle age can be an index of mild cognitive impairment at later years (Nguyen et al. 2014). Given the current lack of effective treatments for AD, and considering AD as a metabolic disease an understanding of the underlying molecular mechanisms occurring during the presymptomatic stages is of considerable importance.

AD is characterized by two histopathological hallmarks: (1) the accumulation of beta‐amyloid protein (Aβ) plaques; and (2) hyper‐phosphorylated tau tangles (Behl and Holsboer 1998). These Aβ peptides have been established as key players in the neuronal degradation that occurs in AD (Gouras et al. 2005), and plays a fundamental role in the mechanisms of early progression of AD (Selkoe 2004; Gandy 2005). Despite an abundance of information concerning AD pathophysiology, the initial events that trigger Aβ plaque formation are poorly understood. Aβ originates from a large type 1 transmembrane protein, known as the amyloid precursor protein (APP) (Selkoe 2001). The β‐secretase pathway is initiated by beta‐site amyloid precursor protein cleaving enzyme 1 (BACE1), the rate‐limiting protease enzyme in the production of Aβ peptides.

Diet‐induced obesity has been implicated in accelerating Aβ production in the development of AD (Lester‐Coll et al. 2006; Verdelho et al. 2007; Maesako et al. 2012a,b, 2013; Bhat and Thirumangalakudi 2013). For example, high‐fat feeding of wild‐type C57BL6/J mice has resulted in increased brain BACE1 and APP protein content, as well as beta‐amyloid peptides (Thirumangalakudi et al. 2008; Puig et al. 2012). Work from our group has demonstrated that high‐fat feeding of wild‐type C57BL6/J mice results in an increase in BACE1 activity (Macpherson et al. 2015). Furthermore, several rodent models of diabetes (genetic, diet and streptozotocin induced) results in increased BACE1 content and beta‐amyloid generation in the brain (Li et al. 2007; Zhang et al. 2009). Similarly, diet‐induced obesity has been shown to accelerate beta‐amyloid pathology in transgenic mouse models of AD (Maesako et al. 2012b, 2013; Bhat and Thirumangalakudi 2013). Sprague Dawley rats fed a high‐sugar and high‐fat diet for 12 months exhibited increased brain beta‐amyloid peptides and phosphorylated tau as early as 6 months (Niu et al. 2016). These studies demonstrate that obesity can promote AD‐like neuropathology, however these studies have examined the effects in a number of different ages and the underlying mechanisms and pathways that contribute to this complex neurodegenerative disease in the early stages remain unclear.

Alterations in inflammatory/cellular stress pathways (mitogen‐activated protein kinases, MAPK), insulin signaling, and markers of energetic stress (5′ adenosine monophosphate – activated protein kinase, AMPK) have all been implicated in AD and the amyloidogenic pathway. The MAPK pathways (extracellular signal‐regulated kinase (ERK), p38, and c‐Jun N‐terminal kinase (JNK)) are activated by extracellular and intracellular stimuli related to stress. MAPKs are serine‐threonine kinases that mediate cellular signaling associated with various cellular activities, which include, cell proliferation, differentiation, survival, and apoptosis (Chang and Karin 2001). The activated MAPK pathways are thought to contribute to AD pathogenesis through various mechanisms including neuronal apoptosis (Hashimoto et al. 2003; Puig et al. 2004), increased BACE1 expression (Tamagno et al. 2009), and the phosphorylation of APP by JNK (Muresan and Muresan 2007; Colombo et al. 2009). Given this link between MAPK pathways and AD, studies examining brain MAPK pathways with obesity are scarce and have inconsistent results. For example, previous work demonstrated that both ERK and p38 phosphorylation were reduced in 24‐month‐old Fischer rats compared to 6‐ and 12‐month‐old rats (Zhen et al. 1999), while a recent study examined diet‐induced obesity and glucose intolerance in mice, demonstrated an increase in ERK phosphorylation and a trend for increased p38 phosphorylation (Macpherson et al. 2015). A 1 year high‐sugar high‐fat diet resulted in increases in beta‐amyloid content along with increased phosphorylated JNK in the hippocampus of rats as early as 6 months (Niu et al. 2016), however an understanding of the temporal relationship between these outcomes is lacking.

Obesogenic diets often result in insulin resistance and a state of energetic stress in peripheral tissues and evidence indicates that obesity also alters brain insulin signaling and in turn energy status. Aberrant control of insulin signaling through the PI3‐kinase and protein kinase B (Akt) has been demonstrated in brain tissue from human AD patients as well as in animal brains of diet‐induced obesity. In human AD samples, Griffin et al. (2005) found increased Akt phosphorylation with significant increases in the phosphorylation of several Akt substrates, including glycogen synthase kinase (GSK). This is important, as GSK has been directly implicated in the progression of AD. Rodent work has demonstrated an ~2‐fold increase in Akt phosphorylation in prefrontal cortex samples from young male C57BL6 mice (~16 weeks of age) following a high‐fat diet (Macpherson et al. 2015) as well as aged mice following 5 months of high‐fat feeding (Muller et al. 2008). Human brains from AD patients demonstrate abnormally activated AMPK (Vingtdeux et al. 2011; Ma et al. 2014) and this thought to be a marker of perturbed brain energy metabolism. The influence of aging and/or diet on brain AMPK activity is poorly understood. Few studies have focused on the direct effect of aging or obesogenic diet on AMPK activity (as indicated by phosphorylation of Thr172), and many of them are contradictory to previous findings. Previous work has demonstrated a decrement in AMPK activation with age in 28‐month‐old Fisher 344 male rats, however the mechanisms remain unknown (Reznick et al. 2007). It is thought that these age associated decreases in AMPK activity may contribute to mitochondrial dysfunction (Jornayvaz and Shulman 2010), which has been associated with the pathogenesis of AD. However, previous work has determined that there is an elevation of the phosphorylation of AMPK in 12‐month‐old AD mouse model (APP_swe_/PS1_d9E_) when compared to wild type (Ma et al. 2014). Further investigation is needed to accurately determine how aging and a high‐fat diet influence the activity and expression of AMPK.

The purpose of this study is to examine the effects of an obesogenic diet on brain metabolic changes in adult male C57BL/6J mice as they transition from adulthood to middle age. Specifically, this study examined markers of inflammation/cellular stress, insulin signaling and energetic stress, all of which have been shown to have implications in the progression of AD. We hypothesize that the consumption of a high‐fat high‐sucrose diet will exacerbate the effects of aging on the metabolic pathways related to the progression of AD.

## Methods and Materials

### Materials

Horseradish peroxidase‐conjugated donkey anti‐rabbit and goat anti‐mouse IgG secondary antibodies were from Jackson ImmunoResearch Laboratories (Westgrove,PA). Molecular weight marker, reagents and nitrocellulose membranes for SDS‐PAGE were acquired from Bio‐Rad (Mississauga, ON). Western lightning Plus‐ELC (PerkinElmer, 105001EA). Antibodies for GAPDH (#2118S), BACE1 (#5606P), AMPK (#2793S), pAMPK (#2531S), AKT (#4685S), pAKT S473 (#4058S), pAKT Thr172 (#2531S), ERK 1/2 (#4695S), pERK 1/2 (#9101S), p38 (#9212S), p‐p38 (#9211S), JNK (#9252S), pJNK (#4671S), GSK‐3β (#9315S) and pGSK‐3β (#5558S) were from Cell Signaling (Danvers, MA, USA) and Vinculin (#05‐386) was from Milipore. All other sources are listed throughout the text.

### Animals

Experimental protocols were approved by the Brock University Animal Care Committee (file #15‐06‐01) and are in compliance with the Canadian Council on Animal Care. Animals and the experimental design are those from a recent study published from Brock University (Bott et al. 2017). Male C57BL/6J mice (19‐weeks; 26.9 ± 1.9 g, *n *= 35) were purchased from The Jackson Laboratory (Bar Harbor, Maine, USA) and allowed to acclimatize for 5 days in the Brock University Comparative Biosciences Facility. During acclimatization, mice were fed standard chow (2014 Teklad global 14% protein rodent maintenance diet, Harlan Tekland, Mississauga, ON). Following acclimatization, mice were randomized to baseline (BSL, *n *= 11), control diet and aging (AGE, *n *= 12), or high‐fat‐sucrose diet in conjunction with aging (AGE‐HFS, *n *= 12). All mice were kept on a 12‐h light: 12‐h dark cycle and had ad libitum access to food and water through the entirety of the study.

### Experimental design

Baseline measures of all mice (*n *= 35) were conducted at 20 weeks of age and included body mass measurements and in vivo *μ*CT scans for body composition (%fat mass, %lean mass). Following baseline measurements, the BSL group (*n *= 11) was euthanized at 22 weeks of age. The AGE (*n *= 12, diet TD.94048, Harlan Teklad, Mississauga, ON) and the AGE‐HFS groups (AGE‐HFS, *n *= 12, diet TD.150448, 45% kcal fat, Harlan Teklad, Mississauga, ON) were switched to their corresponding diets for the 13‐week diet and aging intervention. Harlan Teklad Diet assisted in developing diets that were consistent with previous high fat studies (as previously outlined in detail by (Bott et al. 2017)). Briefly, the control diet (AGE) consisted of 13.7% kcal protein, 75.9% kcal carbohydrates, 10.3% fat and contained 3.6 kcal/g energy. The HFS diet consisted of 13.8% kcal protein, 40.8% kcal carbohydrates, 45.3% fat and contained 4.6 kcal/g energy. To account for the higher energy per quantity of HFS diet, protein and micronutrients were adjusted accordingly, to provide a similar level of these nutrients on the basis of energy (Buettner et al. 2007; Bott et al. 2017). These diet were phytoestrogen free. Body composition measurements were taken longitudinally at 20, 24, 28, and 32 weeks of age, the in vivo body scans were completed using the Bruker Skyscan 1176 *μ*CT (Kontich, Belgium). As well, body weight was measured three times a week using a pan balance throughout the duration of the study. After the 13‐week diet/age intervention, the remaining groups (AGE and AGE‐HFS) were then euthanized for tissue collection.

### Tissue collection

Mice were anaesthetized with 5% isoflurane (CDMV). Mice were euthanized by exsanguination by severing the aorta and vena cava. Collection of brain specific regions (left and right prefrontal cortex and hippocampus) were dissected according to Spijker (2011). Briefly, surgical scissors were used to remove the head with a cut posterior to the ears and a midline incision was made to remove the skin to expose the skull. A small incision was then made at the base of the parietal bone and then cut through to the most anterior part of the skull (i.e., frontal bone). Forceps were then used to peel back and break off both sides of the parietal bone. Once the brain was uncovered, curved surgical scissors (closed) were slid underneath the posterior part of the brain and gently lifted up. The scissors were then used to sever the cranial nerves, freeing the brain. Once the brain was removed from the skull, a surgical scalpel was used to dissect the brain into the left and right hemispheres. Once isolated into the respective sides, the left and right prefrontal cortex and hippocampus were removed. Samples were snap frozen in liquid nitrogen and stored at −80°C for Western blotting analysis.

### Western blotting

Samples were homogenized (FastPrep®, MP Biomedicals, Santa Ana, CA) in 20 volumes of RIPA lysis buffer (abcam, ab15603) supplemented with protease (Sigma‐Aldrich, 11836170001) and phosphatase inhibitors (Sigma‐Aldrich, 04906845001). The homogenized samples were placed on a shaker in a 4°C fridge for 20 min to reduce foam accumulation. Homogenized samples were then centrifuged at 4°C (15 min @ 10,000*g*), after which the supernatant was collected and protein concentration was determined using a Bicinchoninic acid assay (Sigma‐Aldrich ‐ B9643, VWR – BDH9312). The samples were prepared to contain equal concentrations of protein in 2x Laemmli buffer and placed in a dry bath at 100°C for 5 min. Twenty micrograms of protein were loaded and separated on 10% SDS‐PAGE gels for 90 min at 120 V. Proteins were then wet‐transferred onto nitrocellulose membrane at 100 V for 60 min. Membranes were blocked in Tris buffered saline/0.1% Tween 20 (TBST) with 5% non‐fat powdered milk for 1 h at room temperature. The appropriate primary antibody (1:1000 ratio) was then applied and left to incubate shaking overnight at 4°C. Following primary incubation, the membrane was washed with TBST 3 × 5 min and then incubated with the corresponding secondary antibody conjugated with horseradish peroxidase (1:2000 ratio) for 1 h at room temperature. Signals were detected using enhanced chemiluminesence and were subsequently quantified by densitometry using a FluorChem HD imaging system (Alpha Innotech, Santa Clara, CA).

### Statistical analysis

Differences in protein content and phosphorylation were determined using one‐way ANOVA followed by a Tukey's post hoc test. A Shapiro‐Wilk test for normality was conducted and in cases where data were not normally distributed, the data was logarithmically transformed. A value of *P *< 0.05 was considered significant. All data are reported as mean ± SEM.

## Results

### Body weight and composition

Data for body weight and body composition have previously been reported (Bott et al. 2017). Briefly, prior to the dietary intervention there were no differences in body weight between groups (BSL, AGE, AGE‐HFS). Following the 13‐week diet/age intervention, the AGE‐HFS group had a body mass that was 30% higher than the AGE group (*P *= 0.0001). Both AGE and AGE‐HFS groups had an increase in fat mass (*P *= 0.0001), however AGE‐HFS had a greater increase (79%) when compared to AGE (51%; *P *= 0.0001). A decline in lean mass was observed in both AGE and AGE‐HFS (*P *= 0.0001), however AGE‐HFS had a greater decline in lean mass (46%) when compared to AGE (13%; *P *= 0.001). The results demonstrate the effectiveness of the HFS diet to induce a state of obesity.

### Markers of inflammation/cellular stress

**Figure 1:**
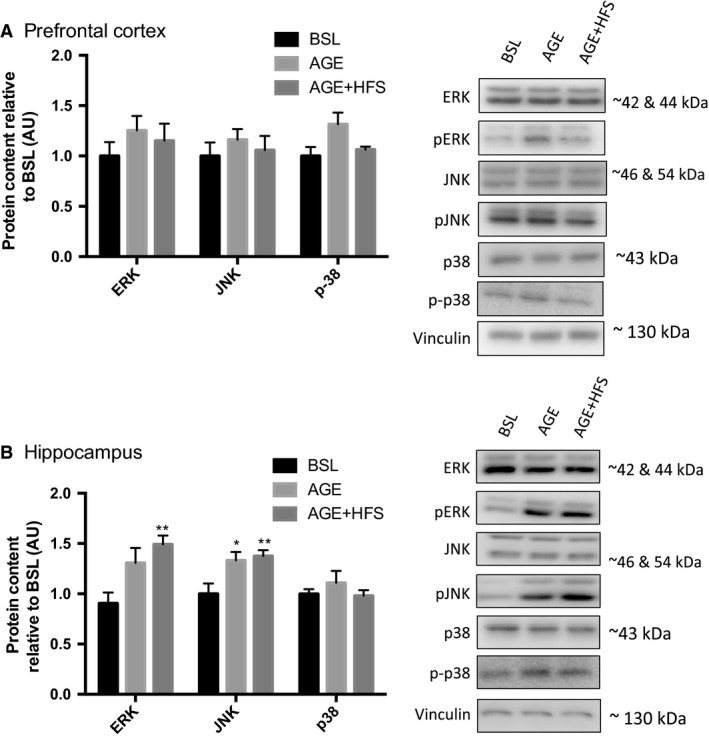
Changes in p38, ERK, and JNK content in the prefrontal cortex (A) and the hippocampus (B) expressed as a ratio of phosphorylated/total in p38, ERK, and JNK. *; Significantly different from BSL (*P *< 0.05). **; Significantly different from BSL (*P *< 0.005). Representative western blots of each protein on the right. All data are reported as mean ± SEM.

The MAPK pathway is thought to be one of the major pathways involved in the progression of AD due to their role in various mechanisms; for example, increased neuronal apoptosis, increased activation of BACE1, and phosphorylation of APP (Hashimoto et al. 2003; Marques et al. 2003; Puig et al. 2004). Changes in total and phosphorylated content of each of the MAPK sub‐families were examined. There were no differences in total p38, ERK, or JNK protein content across all groups in the prefrontal cortex and the hippocampus. There were no differences between groups for pERK, pJNK, or phospho‐p38 in the prefrontal cortex (Fig. 1A). In the hippocampus, there was significantly higher pERK content in the AGE‐HFS group compared to BSL and AGE (*P *= 0.02). Both the AGE and AGE‐HFS groups had higher pJNK protein content compared to BSL (Fig. 1B) (*P *= 0.005).

### Markers of insulin resistance

**Figure 2:**
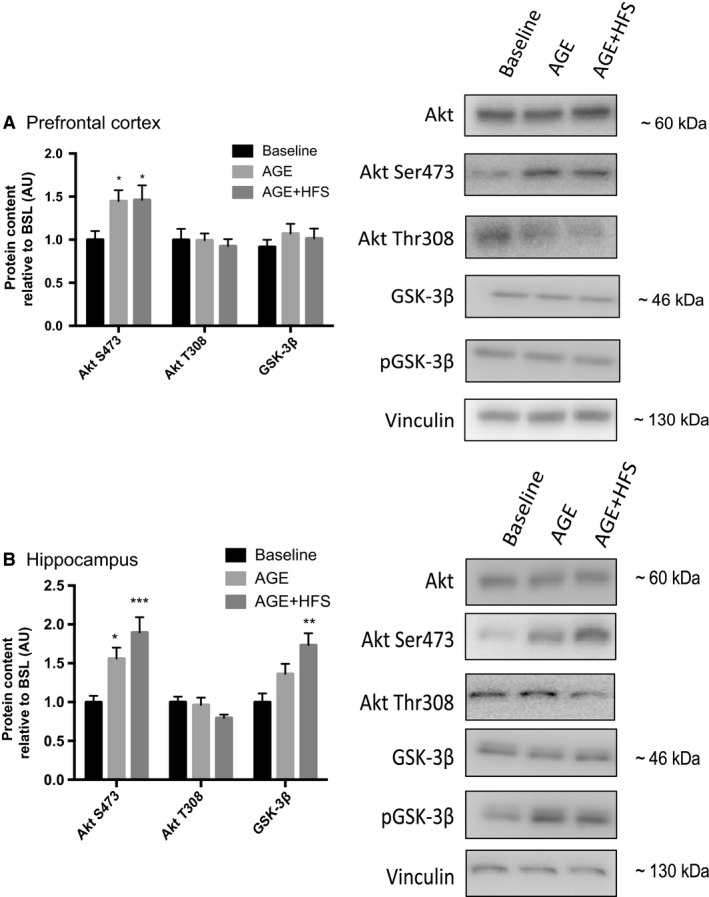
Changes in Akt and GSK‐3β content in the prefrontal cortex (A) and the hippocampus (B) expressed as a ratio of phosphorylated/total in GSK‐3β, Akt Thr308 and Akt Ser 473. *Significantly different from BSL (*P *< 0.05). **; Significantly different from BSL (*P*<0.005). ***; Significantly different from BSL (*P *< 0.0005). Representative western blots of each protein on the right. All data are reported as mean ± SEM.

Evidence suggests that obesity‐related diseases, due to insulin resistance, contribute to neurodegenerative processes such as AD (Bigornia et al. 2012; Candeias et al. 2012). In both the prefrontal cortex and the hippocampus no differences in total Akt or GSK‐3β protein content were observed. The prefrontal cortex demonstrated higher Akt serine 473 phosphorylation in both AGE and AGE‐HFS groups compared to BSL (*P *= 0.03) with no differences in Akt threonine 308 phosphorylation or GSK‐3β phosphorylation (Fig. 2A). The hippocampus demonstrated higher Akt serine 473 phosphorylation in both AGE and AGE‐HFS groups compared to BSL (*P *= 0.0008) with no differences in Akt threonine 308 phosphorylation. Furthermore, in the hippocampus there was higher GSK‐3β phosphorylation in the AGE‐HFS group compared to BSL (Fig. 2B) (*P *= 0.002).

### Markers of energetic stress

**Figure 3:**
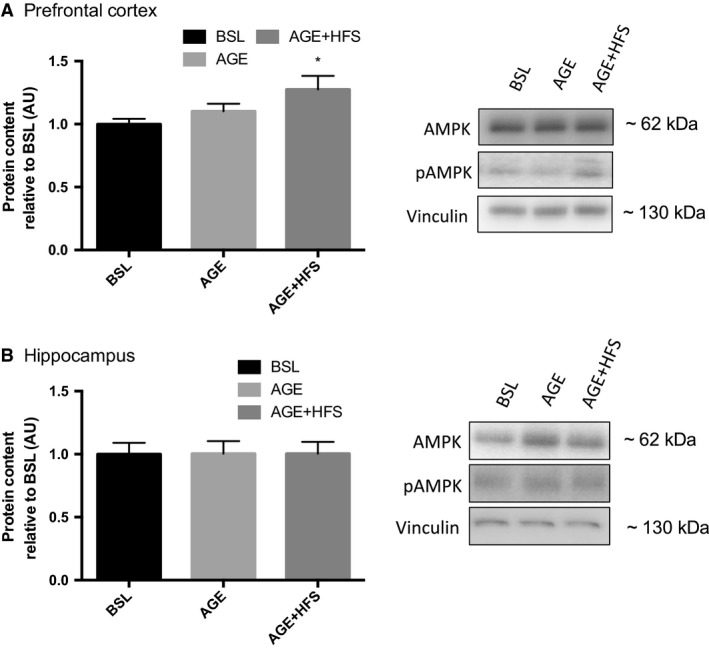
Changes in AMPK content in the prefrontal cortex (A) and the hippocampus (B) expressed as a ratio of phosphorylated/total in AMPK and pAMPK. *; Significantly different from BSL (*P *< 0.05). Representative western blots of each protein on the right. All data are reported as mean ± SEM.

AMPK is a marker of perturbed brain energy metabolism and is abnormally activated in AD (Vingtdeux et al. 2011; Ma et al. 2014). Further AMPK has been found to increase BACE1 protein content and activity (Chen et al. 2009). In the prefrontal cortex there were no differences in total AMPK content between experimental groups; however, there was a higher phosphorylated AMPK content in the AGE‐HFS compared to BSL and AGE (*P *= 0.045) (Fig. 3A). In the hippocampus, there were no significant differences in either total or phosphorylated AMPK (Fig. 3B).

### Amyloid precursors protein processing

**Figure 4:**
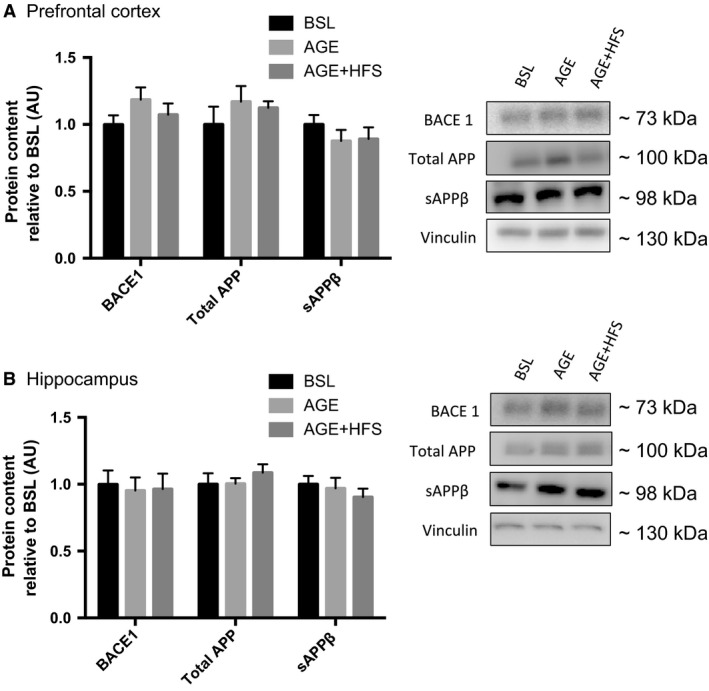
Western blot analysis of changes in BACE1 content and APP peptides in the prefrontal cortex, (A) and hippocampus (B). No significance differences detected. Representative western blots of each protein on the right. All data are reported as mean ± SEM.

BACE1 is considered to be a biomarker for detection and prediction of AD (Hampel and Shen 2009). To examine the effects of aging and aging with a high‐fat/sucrose diet on markers of early AD progression, changes in BACE1, total APP and sAPPβ protein content were measured. In the prefrontal cortex (Fig. 4A), there were no statistically significant changes in BACE1 content, total APP or sAPPβ in either group. In the hippocampus, no changes in BACE1 content, total APP or sAPPβ were detected (Fig. 4B).

## Discussion

This study provides novel insight into the early alterations that occur in the brain in response to a HFS diet during the transition from adulthood to middle age in male mice. Our results demonstrate that alone the transition into middle age results in higher hippocampal JNK and Akt Ser473 phosphorylation and that aging during this period while consuming a HFS diet results in even higher hippocampal JNK and Akt Ser473 phosphorylation as well as ERK and GSK‐3β phosphorylation. Furthermore, in the prefrontal cortex the HFS diet resulted in increased AMPK phosphorylation indicative of energetic stress. These results highlight neuroinflammation/stress, altered brain insulin signaling, and increased energetic stress as important brain alterations with diet‐induced obesity in middle age. This work advances our current knowledge of the underlying mechanisms and pathways that may lead to AD related pathologies in the early stages of the disease. Additional novel results from this work demonstrate region specific differences between the prefrontal cortex and hippocampus in response to aging with a HFS diet, indicating that the disease pathology is not uniform throughout the brain.

Previous work has demonstrated that MAPK signaling is activated in neuronal populations in individuals with AD. Specifically, work examining brain samples from AD patients has demonstrated that neurons and dystrophic neurites have higher JNK (Shoji et al. 2000; Zhu et al. 2001b), p38 (Hensley et al. 1999; Zhu et al. 2000), and ERK activation (Perry et al. 1999; Ferrer et al. 2001a). Here, our results show that aging from adulthood to middle age alone resulted in higher JNK phosphorylation in the hippocampus, while aging with the consumption of a HFS diet resulted in even higher ERK and JNK phosphorylation in the hippocampus. This is interesting as the simultaneous activation of ERK and JNK is thought to represent one of the earliest events in the disease pathogenesis that precipitates further alterations (Zhu et al. 2001a). An examination of MAPKs in brain tissue from post‐mortem patients with varying levels of neurodegeneration, from limited pathology to severe AD, revealed that in control cases without any pathology either ERK alone or JNK alone were phosphorylated, whereas in samples demonstrating limited to full AD pathology both ERK and JNK were simultaneously activated (Zhu et al. 2001a). The results of the present study demonstrate similar findings with the transition from adulthood to middle age resulting in higher hippocampal phosphorylation of JNK while the addition of a HFS diet during this transition results in an increase in both ERK and JNK phosphorylation. This persistent activation of JNK and ERK with the HFS indicates that the hippocampus is under chronic inflammatory stimulation and cellular stress and that this may result in the progression of neurodegeneration and AD‐like pathologies. Our results are further corroborated by earlier studies suggesting that obesity/high fat diets lead to chronic inflammation (Spielman et al. 2014) and increased release of interleukin 1β and TNFα, which are known activators of the MAPKs (Davis et al. 2000; Kim and Choi 2010). These kinases may provide a link to the initiation of AD pathologies. For example, ERK is one of the kinases known to phosphorylate tau and has been shown to be associated with neurofibrillary tangles and senile plaques (Ferrer et al. 2001b). Furthermore, abnormal ERK activation in the hippocampus may impair hippocampal function and contribute to memory deficits in AD patients (Sun and Nan 2017). Our results add to the current literature by providing novel insights into the temporal relationship of these kinases to one another in response to a HFS diet. Further work is needed to examine the relationship to more downstream events related to the pathogenesis of AD. Together this information may help to design strategies that can specifically attenuate ERK and/or JNK promoted neuronal pathologies.

In addition to increased MAPK activity, our results show that transitioning into middle age resulted in increased hippocampal Akt serine 473 phosphorylation, while aging with the HFS diet resulted in increased Akt serine 473 phosphorylation in both the prefrontal cortex and the hippocampus. Brain tissue from post‐mortem patients have also demonstrated increased Akt phosphorylation in both hippocampal and cortical neurons (Pei et al. 2003; Rickle et al. 2004; Griffin et al. 2005). Pei et al. (2003) detected increased Akt threonine 308 phosphorylation associated with varying levels of neurodegeneration, while other studies have demonstrated increased Akt serine 473 phosphorylation in models of AD (Rickle et al. 2004; Griffin et al. 2005; Bhaskar et al. 2009; Kothari et al. 2017). Together, these results would indicate possible constitutive activation of Akt in AD as well as in the early stages of diet‐induced obesity associated neurodegeneration. Activation of Akt is initiated by insulin in a biphasic manner, first by phosphorylation at threonine 308 and then at serine 473. Threonine 308 phosphorylation plays an important role in glucose transport and cell survival, while serine 473 exerts potent inhibitory effects on insulin receptor activity (Tian 2005). Prolonged activation of Akt leads to serine 473 phosphorylation and the negative feedback loop that inhibits insulin signaling (Zhao et al. 2008). The observed increase in Akt Ser473 phosphorylation in the current study may represent insulin resistance in the brain and would have broad consequences for brain function. Akt signaling is a central focus for insulin signaling and the modulation of several cellular processes, including metabolism, growth and proliferation (Brazil et al. 2004). Persistent elevation of Akt serine 473 phosphorylation in brain appears undesirable as abnormally enhanced serine 473 phosphorylation is associated with memory deficits (Dou et al. 2005) and is evident in AD brain (Rickle et al. 2004; Griffin et al. 2005).

In addition to increased inflammatory/cellular stress markers and altered insulin signaling, the AGE‐HFS group also demonstrated increased phosphorylation of AMPK in the prefrontal cortex. These findings support the hypothesis that aging and aging superimposed with a HFS diet increase neural energetic stress, and are in agreement with previous studies (Vingtdeux et al. 2011; Ma et al. 2014; Macpherson et al. 2015). There were no changes in AMPK phosphorylation in the hippocampus thus indicating regional differences in the response to an obesogenic diet. The different responses of the prefrontal cortex and hippocampus to aging and the HFS may be due to differences in energy metabolism between the two regions or it may indicate a different time course in the progression of disease. Further work is needed to examine these regional differences.

## Conclusions

This study provides novel information in relation to the mechanistic link between obesity and the transition from adulthood into middle age and signaling cascades that may be related to AD pathology later in life. Our results demonstrate that aging combined with a HFS diet exacerbates the effects of aging on inflammation/stress (ERK and JNK phosphorylation) in the hippocampus, and energetic stress in the prefrontal cortex. Such adaptations in the prefrontal cortex versus hippocampal regions suggest that disease pathology is not uniform throughout the brain. Furthermore, the HFS diet resulted in higher phosphorylation of Akt Ser473 in both the prefrontal cortex and hippocampus. These results add to our basic understanding of the pathways involved in the early progression of AD pathogenesis and demonstrate the negative effects of a HFS diet on both the prefrontal cortex and hippocampal regions.

## Conflict of Interest

There are no conflicts of interest to disclose.

## References

[phy213729-bib-0001] Behl, C. , and F. Holsboer . 1998 Oxidative stress in the pathogenesis of Alzheimer's disease and antioxidant neuroprotection. Fortschr. Neurol. Psychiatr. 66:113–121. 9565761 10.1055/s-2007-995246

[phy213729-bib-0002] Bhaskar, K. , M. Miller , A. Chludzinski , K. Herrup , M. Zagorski , and B. T. Lamb . 2009 The PI3K‐Akt‐mTOR pathway regulates Abeta oligomer induced neuronal cell cycle events. Mol. Neurodegener. 4:14. 19291319 10.1186/1750-1326-4-14 PMC2663563

[phy213729-bib-0003] Bhat, N. R. , and L. Thirumangalakudi . 2013 Increased Tau phosphorylation and impaired brain insulin/IGF signaling in mice fed a high fat/high cholesterol diet. J. Alzheimers Dis. 36:781–789. 23703152 10.3233/JAD-2012-121030 PMC4445975

[phy213729-bib-0005] Bigornia, S. J. , M. G. Farb , M. M. Mott , D. T. Hess , B. Carmine , A. Fiscale , et al. 2012 Relation of depot‐specific adipose inflammation to insulin resistance in human obesity. Nutr. Diabetes 2:e30. 23449529 10.1038/nutd.2012.3 PMC3341707

[phy213729-bib-0006] Bott, K. N. , W. Gittings , V. A. Fajardo , B. J. Baranowski , R. Vandenboom , P. J. Leblanc , et al. 2017 Musculoskeletal structure and function in response to the combined effect of an obesogenic diet and age in male C57BL/6J mice. Mol. Nutr. Food Res. 61 https://doi.org/10.1002/mnfr.201700137 10.1002/mnfr.201700137 28556515

[phy213729-bib-0007] Brazil, D. P. , Z. Z. Yang , and B. A. Hemmings . 2004 Advances in protein kinase B signalling: AKTion on multiple fronts. Trends Biochem. Sci. 29:233–242. 15130559 10.1016/j.tibs.2004.03.006

[phy213729-bib-0008] Buettner, R. , J. Scholmerich , and L. C. Bollheimer . 2007 High‐fat diets: modeling the metabolic disorders of human obesity in rodents. Obesity (Silver Spring) 15:798–808. 17426312 10.1038/oby.2007.608

[phy213729-bib-0010] Candeias, E. , A. I. Duarte , C. Carvalho , S. C. Correia , S. Cardoso , R. X. Santos , et al. 2012 The impairment of insulin signaling in Alzheimer's disease. IUBMB Life 64:951–957. 23129399 10.1002/iub.1098

[phy213729-bib-0011] Chang, L. , and M. Karin . 2001 Mammalian MAP kinase signalling cascades. Nature 410:37–40. 11242034 10.1038/35065000

[phy213729-bib-0012] Chen, Y. , K. Zhou , R. Wang , Y. Liu , Y. D. Kwak , T. Ma , et al. 2009 Antidiabetic drug metformin (GlucophageR) increases biogenesis of Alzheimer's amyloid peptides via up‐regulating BACE1 transcription. Proc. Natl Acad. Sci. USA 106:3907–3912. 19237574 10.1073/pnas.0807991106 PMC2656178

[phy213729-bib-0013] Colombo, A. , A. Bastone , C. Ploia , A. Sclip , M. Salmona , G. Forloni , et al. 2009 JNK regulates APP cleavage and degradation in a model of Alzheimer's disease. Neurobiol. Dis. 33:518–525. 19166938 10.1016/j.nbd.2008.12.014

[phy213729-bib-0014] Davis, S. , P. Vanhoutte , C. Pages , J. Caboche , and S. Laroche . 2000 The MAPK/ERK cascade targets both Elk‐1 and cAMP response element‐binding protein to control long‐term potentiation‐dependent gene expression in the dentate gyrus in vivo. J. Neurosci. 20:4563–4572. 10844026 10.1523/JNEUROSCI.20-12-04563.2000 PMC6772466

[phy213729-bib-0015] Dou, J. T. , M. Chen , F. Dufour , D. L. Alkon , and W. Q. Zhao . 2005 Insulin receptor signaling in long‐term memory consolidation following spatial learning. Learn Mem. 12:646–655. 16287721 10.1101/lm.88005 PMC1356184

[phy213729-bib-0018] Ferrer, I. , R. Blanco , M. Carmona , and B. Puig . 2001a Phosphorylated mitogen‐activated protein kinase (MAPK/ERK‐P), protein kinase of 38 kDa (p38‐P), stress‐activated protein kinase (SAPK/JNK‐P), and calcium/calmodulin‐dependent kinase II (CaM kinase II) are differentially expressed in tau deposits in neurons and glial cells in tauopathies. J. Neural. Transm (Vienna) 108:1397–1415. 11810404 10.1007/s007020100016

[phy213729-bib-0019] Ferrer, I. , R. Blanco , M. Carmona , R. Ribera , E. Goutan , B. Puig , et al. 2001b Phosphorylated map kinase (ERK1, ERK2) expression is associated with early tau deposition in neurones and glial cells, but not with increased nuclear DNA vulnerability and cell death, in Alzheimer disease, Pick's disease, progressive supranuclear palsy and corticobasal degeneration. Brain Pathol. 11:144–158. 11303790 10.1111/j.1750-3639.2001.tb00387.x PMC8098611

[phy213729-bib-0020] Flegal, K. M. , D. Kruszon‐Moran , M. D. Carroll , C. D. Fryar , and C. L. Ogden . 2016 Trends in obesity among adults in the United States, 2005 to 2014. JAMA 315:2284–2291. 27272580 10.1001/jama.2016.6458 PMC11197437

[phy213729-bib-0021] Gandy, S. 2005 The role of cerebral amyloid beta accumulation in common forms of Alzheimer disease. J. Clin. Invest. 115:1121–1129. 15864339 10.1172/JCI25100 PMC1087184

[phy213729-bib-0022] Gouras, G. K. , C. G. Almeida , and R. H. Takahashi . 2005 Intraneuronal Abeta accumulation and origin of plaques in Alzheimer's disease. Neurobiol. Aging 26:1235–1244. 16023263 10.1016/j.neurobiolaging.2005.05.022

[phy213729-bib-0023] Griffin, R. J. , A. Moloney , M. Kelliher , J. A. Johnston , R. Ravid , P. Dockery , et al. 2005 Activation of Akt/PKB, increased phosphorylation of Akt substrates and loss and altered distribution of Akt and PTEN are features of Alzheimer's disease pathology. J. Neurochem. 93:105–117. 15773910 10.1111/j.1471-4159.2004.02949.x

[phy213729-bib-0025] Hampel, H. , and Y. Shen . 2009 Beta‐site amyloid precursor protein cleaving enzyme 1 (BACE1) as a biological candidate marker of Alzheimer's disease. Scand. J. Clin. Lab. Invest. 69:8–12. 18609117 10.1080/00365510701864610

[phy213729-bib-0026] Hashimoto, Y. , O. Tsuji , T. Niikura , Y. Yamagishi , M. Ishizaka , M. Kawasumi , et al. 2003 Involvement of c‐Jun N‐terminal kinase in amyloid precursor protein‐mediated neuronal cell death. J. Neurochem. 84:864–877. 12562529 10.1046/j.1471-4159.2003.01585.x

[phy213729-bib-0028] Hensley, K. , R. A. Floyd , N. Y. Zheng , R. Nael , K. A. Robinson , X. Nguyen , et al. 1999 p38 kinase is activated in the Alzheimer's disease brain. J. Neurochem. 72:2053–2058. 10217284 10.1046/j.1471-4159.1999.0722053.x

[phy213729-bib-0030] Jorm, A. F. , A. E. Korten , and A. S. Henderson . 1987 The prevalence of dementia: a quantitative integration of the literature. Acta Psychiatr. Scand. 76:465–479. 3324647 10.1111/j.1600-0447.1987.tb02906.x

[phy213729-bib-0031] Jornayvaz, F. R. , and G. I. Shulman . 2010 Regulation of mitochondrial biogenesis. Essays Biochem. 47:69–84. 20533901 10.1042/bse0470069 PMC3883043

[phy213729-bib-0032] Kim, E. K. , and E. J. Choi . 2010 Pathological roles of MAPK signaling pathways in human diseases. Biochim. Biophys. Acta 1802:396–405. 20079433 10.1016/j.bbadis.2009.12.009

[phy213729-bib-0034] Kothari, V. , Y. Luo , T. Tornabene , A. M. O'Neill , M. W. Greene , T. Geetha , et al. 2017 High fat diet induces brain insulin resistance and cognitive impairment in mice. Biochim. Biophys. Acta 1863:499–508. 27771511 10.1016/j.bbadis.2016.10.006

[phy213729-bib-0035] Lester‐Coll, N. , E. J. Rivera , S. J. Soscia , K. Doiron , J. R. Wands , and S. M. de la Monte . 2006 Intracerebral streptozotocin model of type 3 diabetes: relevance to sporadic Alzheimer's disease. J. Alzheimers Dis. 9:13–33. 16627931 10.3233/jad-2006-9102

[phy213729-bib-0036] Li, Z. G. , W. Zhang , and A. A. Sima . 2007 Alzheimer‐like changes in rat models of spontaneous diabetes. Diabetes 56:1817–1824. 17456849 10.2337/db07-0171

[phy213729-bib-0037] Ma, T. , Y. Chen , V. Vingtdeux , H. Zhao , B. Viollet , P. Marambaud , et al. 2014 Inhibition of AMP‐activated protein kinase signaling alleviates impairments in hippocampal synaptic plasticity induced by amyloid beta. J. Neurosci. 34:12230–12238. 25186765 10.1523/JNEUROSCI.1694-14.2014 PMC4152616

[phy213729-bib-0038] Macpherson, R. E. , P. Baumeister , W. T. Peppler , D. C. Wright , and J. P. Little . 2015 Reduced cortical BACE1 content with one bout of exercise is accompanied by declines in AMPK, Akt, and MAPK signaling in obese, glucose‐intolerant mice. J. Appl. Physiol. (1985), 119: 1097–1104. 26404616 10.1152/japplphysiol.00299.2015 PMC4816412

[phy213729-bib-0039] Maesako, M. , K. Uemura , M. Kubota , A. Kuzuya , K. Sasaki , M. Asada , et al. 2012a Environmental enrichment ameliorated high‐fat diet‐induced Abeta deposition and memory deficit in APP transgenic mice. Neurobiol. Aging 33(1011):e11–e23. 10.1016/j.neurobiolaging.2011.10.028 22197104

[phy213729-bib-0040] Maesako, M. , K. Uemura , M. Kubota , A. Kuzuya , K. Sasaki , N. Hayashida , et al. 2012b Exercise is more effective than diet control in preventing high fat diet‐induced beta‐amyloid deposition and memory deficit in amyloid precursor protein transgenic mice. J. Biol. Chem. 287:23024–23033. 22563077 10.1074/jbc.M112.367011 PMC3391129

[phy213729-bib-0041] Maesako, M. , K. Uemura , A. Iwata , M. Kubota , K. Watanabe , M. Uemura , et al. 2013 Continuation of exercise is necessary to inhibit high fat diet‐induced beta‐amyloid deposition and memory deficit in amyloid precursor protein transgenic mice. PLoS ONE 8:e72796. 24023774 10.1371/journal.pone.0072796 PMC3762856

[phy213729-bib-0043] Marques, C. A. , U. Keil , A. Bonert , B. Steiner , C. Haass , W. E. Muller , et al. 2003 Neurotoxic mechanisms caused by the Alzheimer's disease‐linked Swedish amyloid precursor protein mutation: oxidative stress, caspases, and the JNK pathway. J. Biol. Chem. 278:28294–28302. 12730216 10.1074/jbc.M212265200

[phy213729-bib-0044] Muller, A. P. , M. Cammarota , M. O. Dietrich , L. N. Rotta , L. V. Portela , D. O. Souza , et al. 2008 Different effect of high fat diet and physical exercise in the hippocampal signaling. Neurochem. Res. 33:880–885. 18034303 10.1007/s11064-007-9530-7

[phy213729-bib-0045] Muresan, Z. , and V. Muresan . 2007 The amyloid‐beta precursor protein is phosphorylated via distinct pathways during differentiation, mitosis, stress, and degeneration. Mol. Biol. Cell 18:3835–3844. 17634293 10.1091/mbc.E06-07-0625 PMC1995701

[phy213729-bib-0046] Nguyen, J. C. , A. S. Killcross , and T. A. Jenkins . 2014 Obesity and cognitive decline: role of inflammation and vascular changes. Front Neurosci. 8:375. 25477778 10.3389/fnins.2014.00375 PMC4237034

[phy213729-bib-0047] Niu, L. , D. W. Han , R. L. Xu , B. Han , X. Zhou , H. W. Wu , et al. 2016 A high‐sugar high‐fat diet induced metabolic syndrome shows some symptoms of Alzheimer's Disease in rats. J. Nutr. Health Aging 20:509–513. 27102788 10.1007/s12603-015-0601-1 PMC12879883

[phy213729-bib-0049] Pei, J. J. , S. Khatoon , W. L. An , M. Nordlinder , T. Tanaka , H. Braak , et al. 2003 Role of protein kinase B in Alzheimer's neurofibrillary pathology. Acta Neuropathol. 105:381–392. 12624792 10.1007/s00401-002-0657-y

[phy213729-bib-0050] Perry, G. , H. Roder , A. Nunomura , A. Takeda , A. L. Friedlich , X. Zhu , et al. 1999 Activation of neuronal extracellular receptor kinase (ERK) in Alzheimer disease links oxidative stress to abnormal phosphorylation. NeuroReport 10:2411–2415. 10439473 10.1097/00001756-199908020-00035

[phy213729-bib-0052] Puig, B. , T. Gomez‐Isla , E. Ribe , M. Cuadrado , B. Torrejon‐Escribano , E. Dalfo , et al. 2004 Expression of stress‐activated kinases c‐Jun N‐terminal kinase (SAPK/JNK‐P) and p38 kinase (p38‐P), and tau hyperphosphorylation in neurites surrounding betaA plaques in APP Tg2576 mice. Neuropathol. Appl. Neurobiol. 30:491–502. 15488025 10.1111/j.1365-2990.2004.00569.x

[phy213729-bib-0053] Puig, K. L. , A. M. Floden , R. Adhikari , M. Y. Golovko , and C. K. Combs . 2012 Amyloid precursor protein and proinflammatory changes are regulated in brain and adipose tissue in a murine model of high fat diet‐induced obesity. PLoS ONE 7:e30378. 22276186 10.1371/journal.pone.0030378 PMC3261903

[phy213729-bib-0054] Reznick, R. M. , H. Zong , J. Li , K. Morino , I. K. Moore , H. J. Yu , et al. 2007 Aging‐associated reductions in AMP‐activated protein kinase activity and mitochondrial biogenesis. Cell Metab. 5:151–156. 17276357 10.1016/j.cmet.2007.01.008 PMC1885964

[phy213729-bib-0055] Rickle, A. , N. Bogdanovic , I. Volkman , B. Winblad , R. Ravid , and R. F. Cowburn . 2004 Akt activity in Alzheimer's disease and other neurodegenerative disorders. NeuroReport 15:955–959. 15076714 10.1097/00001756-200404290-00005

[phy213729-bib-0056] Selkoe, D. J. 2001 Alzheimer's disease results from the cerebral accumulation and cytotoxicity of amyloid beta‐protein. J. Alzheimers Dis. 3:75–80. 12214075 10.3233/jad-2001-3111

[phy213729-bib-0057] Selkoe, D. J. 2004 Cell biology of protein misfolding: the examples of Alzheimer's and Parkinson's diseases. Nat. Cell Biol. 6:1054–1061. 15516999 10.1038/ncb1104-1054

[phy213729-bib-0058] Shoji, M. , N. Iwakami , S. Takeuchi , M. Waragai , M. Suzuki , I. Kanazawa , et al. 2000 JNK activation is associated with intracellular beta‐amyloid accumulation. Brain Res. Mol. Brain Res. 85:221–233. 11146125 10.1016/s0169-328x(00)00245-x

[phy213729-bib-0059] Spielman, L. J. , J. P. Little , and A. Klegeris . 2014 Inflammation and insulin/IGF‐1 resistance as the possible link between obesity and neurodegeneration. J. Neuroimmunol. 273:8–21. 24969117 10.1016/j.jneuroim.2014.06.004

[phy213729-bib-0060] Spijker, S. 2011 Dissection of rodent brain regions. Neuroproteomics, Neuromethods 57:13–26.

[phy213729-bib-0061] Sun, J. , and G. Nan . 2017 The extracellular signal‐regulated kinase 1/2 pathway in neurological diseases: A potential therapeutic target (Review). Int. J. Mol. Med. 39:1338–1346. 28440493 10.3892/ijmm.2017.2962 PMC5428947

[phy213729-bib-0063] Tamagno, E. , M. Guglielmotto , L. Giliberto , A. Vitali , R. Borghi , R. Autelli , et al. 2009 JNK and ERK1/2 pathways have a dual opposite effect on the expression of BACE1. Neurobiol. Aging 30:1563–1573. 18255190 10.1016/j.neurobiolaging.2007.12.015

[phy213729-bib-0064] Thirumangalakudi, L. , A. Prakasam , R. Zhang , H. Bimonte‐Nelson , K. Sambamurti , M. S. Kindy , et al. 2008 High cholesterol‐induced neuroinflammation and amyloid precursor protein processing correlate with loss of working memory in mice. J. Neurochem. 106:475–485. 18410513 10.1111/j.1471-4159.2008.05415.x PMC3897170

[phy213729-bib-0065] Tian, R. 2005 Another role for the celebrity: Akt and insulin resistance. Circ. Res. 96:139–140. 15692090 10.1161/01.RES.0000156076.17807.1F

[phy213729-bib-0066] Verdelho, A. , S. , Madureira , J. M., Ferro , , H., Chabriat , T., Erkinjuntti , T., Fazekas et al. 2007 Differential impact of cerebral white matter changes, diabetes, hypertension and stroke on cognitive performance among non‐disabled elderly. The LADIS study. J. Neurol. Neurosurg. Psychiatry, 78: 1325–1330. 17470472 10.1136/jnnp.2006.110361 PMC2095587

[phy213729-bib-0067] Vingtdeux, V. , P. Davies , D. W. Dickson , and P. Marambaud . 2011 AMPK is abnormally activated in tangle‐ and pre‐tangle‐bearing neurons in Alzheimer's disease and other tauopathies. Acta Neuropathol. 121:337–349. 20957377 10.1007/s00401-010-0759-x PMC3060560

[phy213729-bib-0068] Zhang, T. , B. S. Pan , B. Zhao , L. M. Zhang , Y. L. Huang , and F. Y. Sun . 2009 Exacerbation of poststroke dementia by type 2 diabetes is associated with synergistic increases of beta‐secretase activation and beta‐amyloid generation in rat brains. Neuroscience 161:1045–1056. 19376202 10.1016/j.neuroscience.2009.04.032

[phy213729-bib-0069] Zhao, W. Q. , F. G. de Felice , S. Fernandez , H. Chen , M. P. Lambert , M. J. Quon , et al. 2008 Amyloid beta oligomers induce impairment of neuronal insulin receptors. FASEB J. 22:246–260. 17720802 10.1096/fj.06-7703com

[phy213729-bib-0070] Zhen, X. , K. Uryu , G. Cai , G. P. Johnson , and E. Friedman . 1999 Age‐associated impairment in brain MAPK signal pathways and the effect of caloric restriction in Fischer 344 rats. J. Gerontol. A Biol. Sci. Med. Sci. 54:B539–B548. 10647963 10.1093/gerona/54.12.b539

[phy213729-bib-0071] Zhu, X. , C. A. Rottkamp , H. Boux , A. Takeda , G. Perry , and M. A. Smith . 2000 Activation of p38 kinase links tau phosphorylation, oxidative stress, and cell cycle‐related events in Alzheimer disease. J. Neuropathol. Exp. Neurol. 59:880–888. 11079778 10.1093/jnen/59.10.880

[phy213729-bib-0072] Zhu, X. , R. J. Castellani , A. Takeda , A. Nunomura , C. S. Atwood , G. Perry , et al. 2001a Differential activation of neuronal ERK, JNK/SAPK and p38 in Alzheimer disease: the ‘two hit’ hypothesis. Mech. Ageing Dev. 123:39–46. 11640950 10.1016/s0047-6374(01)00342-6

[phy213729-bib-0073] Zhu, X. , A. K. Raina , C. A. Rottkamp , G. Aliev , G. Perry , H. Boux , et al. 2001b Activation and redistribution of c‐jun N‐terminal kinase/stress activated protein kinase in degenerating neurons in Alzheimer's disease. J. Neurochem. 76:435–441. 11208906 10.1046/j.1471-4159.2001.00046.x

